# Hunter Island Group Phlebovirus in Ticks, Australia

**DOI:** 10.3201/eid2112.141303

**Published:** 2015-12

**Authors:** Penelope J. Gauci, Jane McAllister, Ian R. Mitchell, Toby D. St. George, Daisy H. Cybinski, Steven S. Davis, Aneta J. Gubala

**Affiliations:** Defence Science & Technology Organisation, Fishermans Bend, Victoria, Australia (P.J. Gauci, J. McAllister, I.R. Mitchell, A.J. Gubala);; Long Pocket Laboratories, Indooroopilly, Queensland, Australia (T.D. St. George, D.H. Cybinski, S.S. Davis);; Department of Primary Industry and Fisheries, Berrimah, Northern Territory, Australia (S.S. Davis)

**Keywords:** Bunyaviridae, phlebovirus, Australia, Albatross Island virus, Hunter Island Group virus, viruses, zoonoses, Australia, ticks, vector-borne infections

**To the Editor:** A recent article described the isolation and subsequent analysis of a tickborne phlebovirus: Hunter Island Group virus (HIGV), associated with an albatross disease event that occurred in 2002 on Albatross Island, 6 kilometers off the northwest coast of Tasmania, Australia (*1*). The authors present HIGV as a novel isolate; however, new data and historical records demonstrate that the virus was originally isolated in 1983. Provisionally named Albatross Island virus (ABIV), the virus was classified as unidentified because of its uniqueness and dissimilarity to any known virus in Australia. ABIV and HIGV were isolated from ticks of the same species, *Ixodes eudyptidis*, collected from the nests of shy albatross (*Thalassarche cauta*) on Albatross Island, the only island inhabited by albatross within the Hunter Island Group Important Bird Area. At the time of collections, many immature albatross were dying. Records from this time indicate that postmortem blood samples were collected from the birds, and subsequent virus neutralization studies conducted soon after demonstrated that 50% of these samples were ABIV positive. Ensuing testing of samples collected in the next 2 years also identified a positive sample from a black noddy in Queensland (Table). ABIV was subsequently sent for testing at the Arbovirus World Reference Laboratory and, more than a decade later, to the Australian Animal Health Laboratory, Commonwealth Scientific and Industrial Research Organisation, where it was identified as a bunyavirus but remained largely uncharacterized.

**Table:** Virus neutralization assay results for Albatross Island virus, Australia

Source (species)	Location/year sample collected	No. positive/no. tested
Magpie goose (*Anseranas semipalmata*)	Northern Territory/1974	0/2
Masked lapwing (*Vanellus miles*)	Northern Territory/1975	0/1
Little corella (*Cacatua sanguinea*)	Northern Territory/1975	0/1
Whimbrel (*Numenius phaeopus*)	Northern Territory/1975	0/8
Cattle egret (*Ardea ibis*)	Gatton, Queensland/1981	0/5
Shy albatross (*Thalassarche cauta*)	Albatross Island/1983	18/36
Black noddy (*Anous minutus*)	Heron Island, Queensland /1985	1/115
Wedge-tailed shearwater (*Ardenna pacifica*)	Heron Island, Queensland /1985	0/46
Domestic chickens (*Gallus gallus*)	Armidale, New South Wales/1979	0/10
Little red flying fox (*Pteropus scapulatus*)	Unknown/unknown	0/5
Seals (unknown)	Macquarie Island/unknown	0/35
Maruspials (various wallabies, kangaroos, possums)	Queensland /Northern Territory/unknown	0/97
Cattle (*Bos taurus*)	Queensland /Northern Territory/1974–1985	0/160
Rusa deer (*Rusa timorensis*)	SE Queensland/1984	0/30

We recently sequenced the genome of ABIV by using high-throughput sequencing and have compiled near complete sequences for the large (L), medium (M), and small (S) segments (GenBank accession nos. KM198925–7). Overall, ABIV shares 99% nt identity with HIGV, and thus they can be considered isolates of the same virus. The translated nucleocapsid and S segment nonstructural proteins of both viruses are identical, and the polymerases and glycoproteins share 99% identity. There are 26 nt changes across the whole genome (1 in S, 8 in M, 17 in L), but only 7 of these translate into an amino acid change (3 in the Gn/Gc polyprotein, 4 in the polymerase protein). Predictive protein analysis indicates that at least 1 of the 3 aa changes occurs in the ectodomain of the Gn protein, which could affect virus–host interactions. Of the remaining changes, 14 are silent mutations and 5 occur in noncoding regions. 

In light of the genomic similarity of these 2 viruses, we suggest that the species name Albatross Island virus encompass both isolates, ABIV and HIGV, thereby representing the name of the original 1983 isolate and the location where both viruses were isolated. These 2 viruses are closely related to 2 tickborne phleboviruses: severe fever with thrombocytopenia syndrome virus, isolated in China (*2*), and Heartland virus, isolated in the United States (*3*). Each of these recently emerged viruses causes severe febrile illness with thrombocytopenia; deaths have been reported from 4 countries. In addition, Malsoor virus (*4*), a phlebovirus recently isolated from bats in India, has been shown to be closely related to severe fever with thrombocytopenia syndrome virus and Heartland virus. At the protein level, the similarity of ABIV to these 3 viruses is as follows: L, 66%–67%; M, 52%–56%; S, 58%–62%.

Deaths of albatross chicks in the Albatross Island colony occur every year; the intensity of these events varies from year to year (*5*). The cause of these events is multifaceted, but fowlpox is believed to be a major factor (*6*). No tests exist to quantify the extent and cause of the problem, although solutions are being pursued (R. Alderman, pers. comm, 2015). Wang et al. were unable to confirm infection of albatross with the HIGV isolate (*1*); however, the results presented here suggest that ABIV does infect albatross. Although infection is not direct evidence of disease, the fact that both isolates were collected from the same albatross colony during disease events almost 2 decades apart should not be neglected. Viral challenge studies would be useful for determining if and how ABIV contributes to disease in these birds.

Birds of the albatross family tend to fly long distances over open water. The geographic range of shy albatross extends from their breeding base in Tasmania to southern Africa (*5*). White-capped albatross (*T. steadi*) reportedly migrate from their breeding base in New Zealand as far as South America and eastward into shy albatross territory (*7*). It is possible to misidentify 1 of these albatross species as the other; indeed, the phylogenetic distinction between these species, once considered the same (*Diomedea cauta*), is controversial. The ease of albatross movement between vast geographic areas could provide an opportunity for intercontinental spread of emerging infectious diseases. Consequently, phleboviruses similar to ABIV may be present in bird populations in the southern areas of Africa and South America.

The need for intensified international investigations to identify genetically related tickborne phleboviruses with zoonotic potential is evident. The opportunity for the distribution of such viruses over a large global area is of concern to public health. Surveillance and investigation on an international level are needed.
